# Identifying the transition to the maturation zone in three ecotypes of *Arabidopsis thaliana* roots

**DOI:** 10.1080/19420889.2017.1395993

**Published:** 2017-12-14

**Authors:** Wendy Cajero Sánchez, Berenice García-Ponce, María de la Paz Sánchez, Elena R. Álvarez-Buylla, Adriana Garay-Arroyo

**Affiliations:** Laboratorio de Genética Molecular, Desarrollo, Evolución y Epigenética de Plantas, Universidad Nacional Autónoma de México, 3er Circuito Exterior junto al Jardín Botánico, Ciudad Universitaria, Ciudad de México, Mexico

**Keywords:** Arabidopsis root growth, Maturation Zone (MZ), root cell quantification

## Abstract

The *Arabidopsis thaliana* (hereafter Arabidopsis) root has become a useful model for studying how organ morphogenesis emerge from the coordination and balance of cell proliferation and differentiation, as both processes may be observed and quantified in the root at different stages of development. Hence, being able to objectively identify and delimit the different stages of root development has been very important. Up to now, three different zones along the longitudinal axis of the primary root of Arabidopsis, have been identified: the root apical meristematic zone (RAM) with two domains [the proliferative (PD) and the transition domain (TD)], the elongation zone (EZ) and the maturation zone (MZ). We previously reported a method to quantify the length of the cells of the meristematic and the elongation zone, as well as the boundaries or transitions between the root domains along the growing part of the Arabidopsis root. In this study, we provide a more accurate criterion to identify the MZ. Traditionally, the transition between the EZ to the MZ has been established by the emergence of the first root-hair bulge in the epidermis, because this emergence coincides with cell maturation in this cell type. But we have found here that after the emergence of the first root-hair bulge some cells continue to elongate and we have confirmed this in three different Arabidopsis ecotypes. We established the limit between the EZ and the MZ by looking for the closest cortical cell with a longer length than the average cell length of 10 cells after the cortical cell closest to the epidermal cell with the first root-hair bulge in these three ecotypes. In *Col-0* and *Ws* this cell is four cells above the one with the root hair bulge and, in the *Ler* ecotype, this cell is five cells above. To unambiguously identifying the site at which cells stop elongating and attain their final length and fate at the MZ, we propose to calculate the length of completely elongated cortical cells counting 10 cells starting from the sixth cell above the cortical cell closest to the epidermal cell with the first root-hair bulge. We validated this proposal in the three ecotypes analyzed and consider that this proposal may aid at having a more objective way to characterize root phenotypes and compare among them.

## Introduction

Plants are sessile organisms that present plastic growth and developmental responses to environmental conditions along their life cycle. Such adjustments are reflected in altered patterns of cell proliferation/differentiation rates that, in turn, underlie plastic plant morphogenetic patterns. In the root apical meristematic zone (RAM), cells are produced at the stem cell niche, proliferate for 4–6 cycles in the proliferation/transition domains and displace the older proliferating cells away from the stem-cell niche towards the elongation zone (EZ) where cells stop proliferating and start elongating anisotropically at very fast rates. Finally, cells attain the maturation zone (MZ) where they differentiate terminally and attain their final length.^1–3^ The processes of cell proliferation and differentiation are occurring simultaneously and there is a balance between them that can be altered by internal or external signals or modulating mechanisms.^4^ In addition, these cellular patterns, are emergent from complex underlying dynamic systems that include genetic and non-genetic components.^5,6^ Hence, plant cell growth and development results from a coordination of cell proliferation and elongation across tissues, because cells from different tissues are symplastically connected.^7,8^

Arabidopsis has one main primary root that is formed during embryogenesis but experience an intense postembryonic growth and development.^9^ This root has become a useful model for studying the coordination between cell proliferation and differentiation during organ morphogenesis, as well as underlying genetic regulatory mechanisms, as in this organ both processes can be observed and quantified at different stages of development at the same time.^7,8,10,11^ This is plausible, among other things, because roots are transparent and readily observable and cell size, cell proliferation rate and cell number can be quantified at different stages of root development along their longitudinal axis at any particular time and at different stages of root development. Finally, the root has relatively few cells and cell types organized in rather stereotypical longitudinal and radial patterns.^12^

The apex of the RAM contains the Stem Cell Niche (SCN) that is formed by four different types of stem cells, also called initials^12^ that yield all the different types of root cells. The initial cells surround an organizer center called the Quiescent Center (QC) formed by four cells with very low mitotic activity. Initial cells are replenished from the QC via asymmetric cell division with one cell remaining at the QC and the other one contributing to the stem cells pool. The QC cells seem to produce short-range signals that are important for maintaining the initial cells in an undifferentiated state and the identity of them depends on signals coming from mature cells towards the plant basis.^13,14^ Once cells enter the proliferation domain (PD) of the meristem they start dividing at very high rates until they are displaced basally and start to divide at lower rates. At the transition domain (TD), before starting to elongate, cells divide at slower rates and have unique physiological properties such as alterations in their cell-wall structure and vacuolization that enables fast length growth at the EZ.^2,3,15^ Finally, in the MZ root cells attain their final fate and size; at this level, root hairs are formed in epidermal cells, and protoxylema vessels are specified.^16^

Recently we proposed a multiple structural change algorithm (MSC) to quantitatively identify the PD, ED and EZ zones and domains of the root and particularly as an objective means to identify the transitions between the PD and the TD and the TD and the EZ.^17^ We did not center our attention in identifying the transition from the EZ to the MZ as this transition was supposed to be easily identified by the appearance of the first bulge of root hair in the root epidermis.^18–21^ Nevertheless, there are some reports that have noted that root hair initiation marks the end of rapid elongation but not the initiation of the maturation zone where cell elongation rates should be equal to zero.^15,22,23^ Moreover, some authors integrate the elongation and maturation zones and consider that the onset of elongation marks the initiation of differentiation, and hence they propose that it is not important to recognize the transition between the elongation and the maturation zones.^3^ Nonetheless, cell wall structure;^15,24^ microtubule orientation^25^ and root hair growth,^15^ -among other cellular traits-, are different between the different root zones.^15,26,27^

As the maximum cell length has been used to analyze the effect that different gene mutations or hormone treatments have on cell behavior at the maturation zone^19–21,28,29^ we consider that it is important to clearly identify the maturation zone as well. In this study we quantified cell elongation along the longitudinal root axis with relation to root hair bulge formation in three Arabidopsis ecotypes. We found that cell elongation continues in all of three ecotypes after the first hair bulge is observed. Hence, we propose to calculate the length of completely elongated cortical cells counting 10 cells starting from the sixth cell after the cortical cell closest to the epidermal first root-hair cell as an indication of the initiation of the maturation root zone.

## Material and methods

### Plant materials and growth conditions

We used three *Arabidopsis thaliana* ecotypes: Columbia (*Col-0*), Wassilewskija (*Ws*) and Landsberg erecta (*Ler-1*) that have contrasting root parameters. Seeds were surfaced sterilized by shaking them for 5 minutes in 100% EtOH, then they were decanted and transferred to a solution of chlorine/SDS 5%/1% for 13 minutes and washed three times in sterile water before sowing. Seeds were stratified for 5 days at 4°C and then sown on vertical plates with 0.2X MS salts, 1% sucrose and 1% agar (unless otherwise indicated). Plants were grown for 6 or 11 dps (days post sowing) under long-day (LD; 16 h light/8 h dark) conditions in growth chambers at 22°C, with a 16-h photo/8-h dark period (110 mEm^−2^ s^−1^).

### Microscopy

To measure root cell size, seedlings were fixed in 50% methanol and 10% acetic acid at 4°C for 24 hours. After fixation, roots were incubated for 40 min in 1% periodic acid at 37°C, washed for three times with distilled water and then seedlings were incubated in a 100 mM sodium bisulfite, 0.15 N hydrochloric acid, and 100 μg/ mL propidium iodide solution for one hour. After that, seedlings were washed again three times with distilled water and placed in DMSO/Glycerol (2%/30%) for two days. Finally, the roots (17 for each ecotype) were soaked in a sodium Iodide solution over the slide, to be observed and quantified under a Nomarski optics using an Olympus BX40 microscope. Confocal images were acquired using an inverted Nikon Ti Eclipse with a dry X20 objective after root tissue was stained with 5 or 10 g μl^−1^ propidium iodide.

### Quantitative analysis

All cell length measures were done using ImageJ software. Seventeen roots per ecotype were observed in each case and quantitative cellular measurements for the different root growth domains and zones were obtained using the multiple structural change algorithm (MSC) program described.^17^ To obtain the length of fully elongated cortical cells we measured 20 cells of seventeen plants starting 1) from the cortical cell closest to the epidermal cell with the first hair bulge or 2) from the sixth cortical cell after the cortical cell closest to the epidermal cell with the first root hair bulge (see Fig. 5 and Table 1). For cell quantification data, an ANOVA was used to compare the ecotypes and Tukeys test (P < 0.05) to perform all pairwise comparisons with the program Graphpad Prism 6.

**Table 1.** Quantitative cellular analysis of roots from three different ecotypes of *Arabidopsis thaliana* (6 dps).

	*Col-0*	*Ler-1*	*Ws*
Number of proliferation domain cells	34 ± 0.835	30 ± 1.57	26 ± 0.85
Number of RAM cells	44 ± 1.44	42 ± 1.71	37 ± 0.93
Proliferation Domain size (µm)	196 ± 6.05	172 ± 11.59	177 ± 3.56
Transition Domain size (µm)	145 ± 7.56	131 ± 4.87	116 ± 4.91
RAM size (µm)	341 ± 8.25	303 ± 14.21	289 ± 6.06
Elongation zone size (µm)	447 ± 13.23	352 ± 15.48	435 ± 17.49
A) Average length of the cortical cell closest to the epidermal cell with			
the first hair bulge (µm)	101.54	85.22	114.96
B) Average of 10 fully elongated cells (µm) starting from the cortical cell			
closest to the epidermal cell with the first root hair bulge	153.81	109.93	144.40
C) Average of 10 fully elongated cells (µm) starting from the cortical cell six cells			
above the cortical cell closest to the epidermal cell with the first root hair bulge	169.50	124.56	155.57
D) Average of 15 fully elongated cortical cells (µm) starting from the cortical cell			
closest to the epidermal cell with the first root hair bulge	158.38	114.91	148.42
E) Average of 15 fully elongated cells (µm) starting from the cortical cell six above			
the cortical cell closest to the epidermal cell with the first root hair bulge	165.85	124.97	157.09
F) Average of 25 fully elongated cells (µm) starting from the cortical cell			
the cortical cell closest to the epidermal cell with the first root hair bulge	159.04	118.36	153.87
G) Average of 25 fully elongated cells (µm) starting from the cortical cell six above			
the cortical cell closest to the epidermal cell with the first root hair bulge	166.87	126.92	162.26
H) Subtraction of the two averages of 10 cells (µm) C-B	15.69	14.63	11.17
I) Subtraction of the two averages of 15 cells (µm) E-D	7.47	10.06	8.67
J) Subtraction of the two averages 25 cells (µm) G-F	7.83	8.56	8.39

## Results and discussion

### Cell sizes in the cortical layer of the root

**Figure 1.**
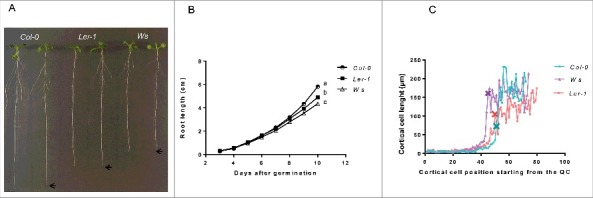
Root growth and cellular length differ among *Col-0, Ler* and *Ws Arabidopsis thaliana* ecotypes. (A) Root length phenotypes of seedlings of *Col-0, Ler* and *Ws* ecotypes at 11 dps (n=30). (B) Root growth curves of *Col-0, Ler* and *Ws* ecotypes grown for 10 days (n=40). The average of the values is shown and the letters show the significant differences between the three ecotypes with a confidence interval ≥ 95%. (C) Cell sizes in the cortical layer of the root (of cell 1 from QC to 25 cells after the first primordial hair cell) **x** show the cortical cell closest the epidermal cell with the first hair bulge of *Col-0, Ler* and *Ws* ecotypes.

**Figure 2.**
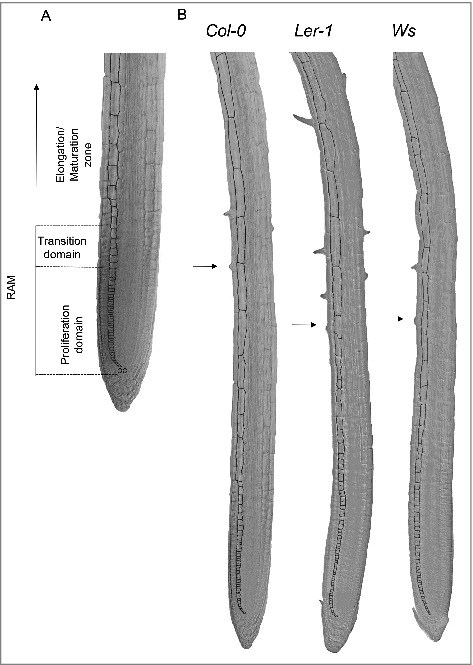
Root cell length of cortical cells is different among *Col-0, Ler* and *Ws Arabidopsis thaliana* ecotypes. The cortical cells perimeter is marked with black to show the cell´s form. A) Root apical meristem (RAM) and elongation zone (EZ) of Arabidopsis *Col-0, Ler* and *Ws* ecotypes. B) Seedling roots from *Col-0, Ler* and *Ws* ecotypes grown for 6 dps. The arrow marks the epidermal cell with the first hair bulge.

**Figure 3.**
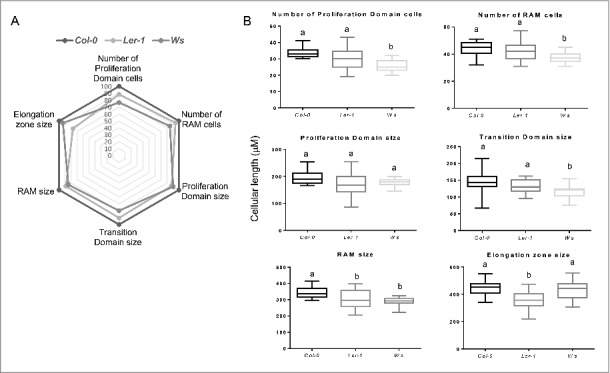
Analysis of different cell parameters along the primary root of the *Col-0, Ler* and *Ws Arabidopsis thaliana* ecotypes. (A) Spider graphical model analysis of different cellular quantifications of the three *Col-0, Ler* and *Ws* ecotypes. The values represents ratio indicators taking *Col-0* as the reference (100%), in all the parameters. B) Box plot from each of the measured parameters, the letters show the significant differences with a confidence interval ≥ 95% for *Col-0, Ler* and *Ws* ecotypes.

We decided to use three different ecotypes of Arabidopsis [Columbia (*Col-0*), Wassilewskija (*Ws*) and Landsberg erecta (*Ler-1*)] because they have different primary root length and also differ in several aspects of the quantitative cellular analysis (Aceves-García et al., 2016) (Fig. 1A and B); this enabled us to show that the quantitative cellular results shown here do not depend on total root length. Since RAM organization is clearly visible in median longitudinal sections of the root (Fig. 2A), we measured the length of the cortical root cells in one file from the quiescent center (QC) up to 25 cells after the cortical cell closest to the epidermal cell with the first hair bulge (**x** in Fig. 1C). With these cell measures, we used the multiple structural change algorithm (MSC) program^17^ to obtain the length and number of cells of the RAM and of the two domains within it, as well as the elongation zone size along the root longitudinal axis. As can be seen in Fig. 3A and 3B, the number of cells in the RAM is different between *Ws* and the two other ecotypes (*Col-0* and *Ler*) and this could be explained as the number of cells in the proliferation domain is the one that is different. Interestingly, the elongation zone size and the length of fully elongated cells are different between the *Ler* ecotype and the other two (*Col-0* and *Ws*) (Fig. 5 and Table 1). Our data suggest, therefore, that the ecotypes primary root length differ among them as a result of a combination of a decreased number of cells in the RAM and shorter final cell length in the MZ, being the proliferation rate more important for root growth in the *Ws* ecotype and the elongation rate in the *Ler* ecotype compared to *Col-0* ecotype.

**Figure 4.**
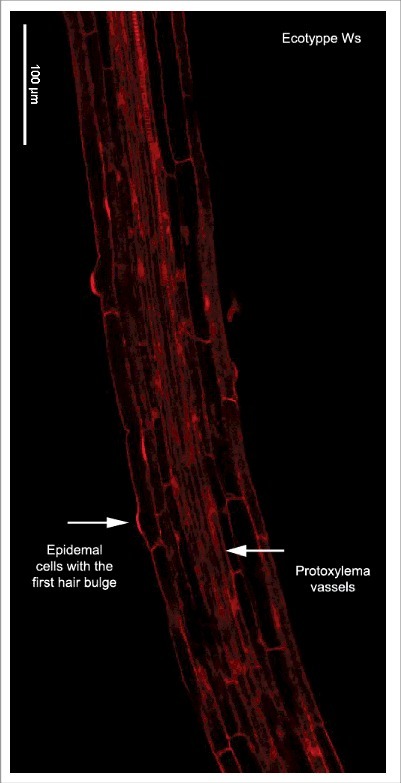
Confocal image of a root showing two markers of differentiation. Root longitudinal optical section of a 7 days after sowing seedling of *Ws* ecotype. The red signal is emitted by propidium iodide that was used as a counterstain and the arrows show the first differentiation vascular and epidermal cell.

**Figure 5.**
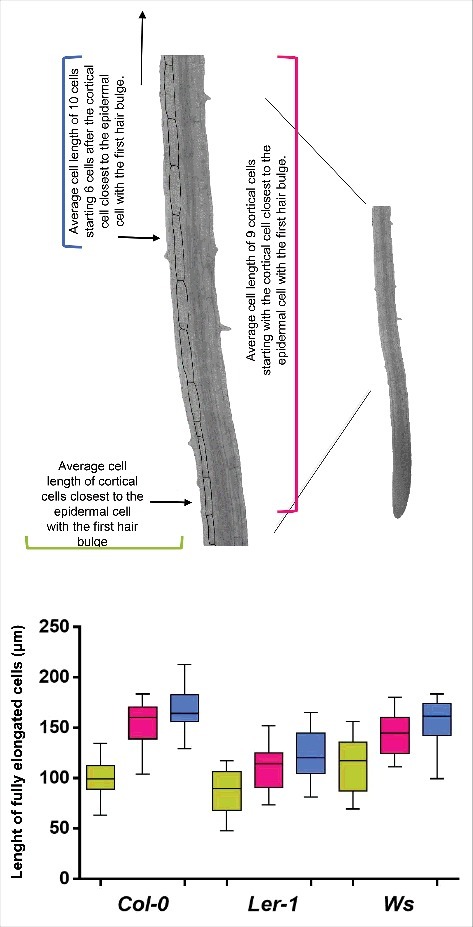
Root cell length of fully elongated cells is different among *Col-0, Ler* and *Ws Arabidopsis thaliana* ecotypes. Elongated cortical cell length of 17 roots of the three ecotypes. Green: average cell length of cortical cells closest to the epidermal cell with the first hair bulge, pink: average cell length of 9 cortical cells starting with the cortical cell closest to the epidermal cell with the first hair bulge and blue: average cell length of 10 cells starting 6 cells after the cortical cell closest to the epidermal cell with the first hair bulge.

In the case of the length of fully elongated cells in the MZ, we used the average of 25 cortical cells beginning with the cortical cell closest to the epidermal cell where the first root hair bulge is observed (arrow in Fig. 2B). Besides, we looked for the protoxylema vessels as a marker of differentiation and, as expected, we found that this marker is on the same area as the marker of differentiation of the epidermal cells (Fig. 4). However, we noticed that, after both of these markers of differentiation are observed, cell elongation continues to occur in the three ecotypes used (see Table 1 and Fig. 5). Surprisingly, nonetheless, there is a big difference between the average cell length of the cortical cells closest to the epidermal cell where the first root hair bulge emerges and the average cell length of fully elongated cortical cells measuring either 10, 15 or 25 cells starting from the cortical cell closest to the epidermal cell with the first hair cell bulge (Fig. 5 and Table 1). Given the importance of unambiguously identifying the transition of the EZ to the MZ, we decided to look for a better way to obtain the average length of completely elongated cells. For this, we averaged the length of 10 cortical cells starting with the cortical cell closest to the epidermal cell with the first hair bulge to obtain the final length of completely elongated cortical cells usually used. Then, we looked for the cortical cell that has a length equal or larger than this average length to assure that we are on the MZ and we found that, in two ecotypes (*Col-0* and *Ws*), this cell is four cells away and in *Ler* is five cells away from the cortical cell closest to the epidermal cell with the first hair bulge (Fig. 5). So, we decided to obtain the length of completely elongated cells counting 10 cells starting six cells above the cortical cell closest to the epidermal cell with the first hair bulge. As expected, the estimated average length of fully elongated cells is different when considering the cortical cell closest to the epidermal cell that coincides with the first root hair bulge, in comparison to using the one that is six cells above and, in all cases, the average length is higher when using the second measurement (Table 1). It is noteworthy that in ecotypes *Col-0* and *Ler*, the difference between these two measures counting 10 cells is almost 16 μm (Table 1) and in the *Ws* ecotype the difference is also quite large: 11μm (Table 1). Finally, when we compared the average length of fully elongated cortical cells using 10, 15 and 25 cortical cells starting from the cortical cell closest to the epidermal cell with the first hair bulge and six cells above, we found that there is a difference in the average cell length of near 8 μm in each ecotype comparing the measures in 15 and 25 cells (Table 1). Therefore, we propose to calculate the length of completely elongated cortical cells counting 10 cells starting from the cortical cell sixth cells above the cortical cell closest to the epidermal cell with the first root-hair bulge as a means to establish the initiation of the mature zone of the Arabidopsis root.

In conclusion, root development varies considerably among Arabidopsis ecotypes. Quantitative and objective comparable methods are necessary in order to gather accurate accounts of root cellular dynamics. These are key for understanding what are the cellular components that underlie contrasting root growth dynamics and patterns.

Arabidopsis roots are organized in three zones along longitudinal axis: meristem, elongation and maturation zone. This study provides an objective and quantitative means to establish the transition from the elongation to the maturation zone in three ecotypes with contrasting primary root lengths and growth patterns. Our data show that the transition between these two zones is clearly defined if we start counting the length of fully cortical elongated cells, counting six cells above the cortical cell closest to the epidermal cell where the first root-hair bulge is produced.
